# Clever Experimental Designs: Shortcuts for Better iPSC Differentiation

**DOI:** 10.3390/cells10123540

**Published:** 2021-12-15

**Authors:** Ryota Yasui, Keisuke Sekine, Hideki Taniguchi

**Affiliations:** 1 Department of Regenerative Medicine, Yokohama City University Graduate School of Medicine, Yokohama 236-0004, Japan; r_yasui@yokohama-cu.ac.jp; 2 Fundamental Research Laboratory, Eiken Chemical Co., Ltd., Tochigi 329-0114, Japan; 3 Laboratory of Cancer Cell Systems, National Cancer Center Research Institute, Tokyo 104-0045, Japan; 4 Division of Regenerative Medicine, Center for Stem Cell Biology and Regenerative Medicine, The Institute of Medical Science, The University of Tokyo, Tokyo 108-8639, Japan

**Keywords:** design of experiments (DOE), induced pluripotent stem cell (iPSC), embryonic stem cell (ESC), mesenchymal stem cell (MSC), hematopoietic stem cell (HSC), Chinese hamster ovary (CHO) cell, cell differentiation

## Abstract

For practical use of pluripotent stem cells (PSCs) for disease modelling, drug screening, and regenerative medicine, the cell differentiation process needs to be properly refined to generate end products with consistent and high quality. To construct and optimize a robust cell-induction process, a myriad of cell culture conditions should be considered. In contrast to inefficient brute-force screening, statistical design of experiments (DOE) approaches, such as factorial design, orthogonal array design, response surface methodology (RSM), definitive screening design (DSD), and mixture design, enable efficient and strategic screening of conditions in smaller experimental runs through multifactorial screening and/or quantitative modeling. Although DOE has become routinely utilized in the bioengineering and pharmaceutical fields, the imminent need of more detailed cell-lineage specification, complex organoid construction, and a stable supply of qualified cell-derived material requires expedition of DOE utilization in stem cell bioprocessing. This review summarizes DOE-based cell culture optimizations of PSCs, mesenchymal stem cells (MSCs), hematopoietic stem cells (HSCs), and Chinese hamster ovary (CHO) cells, which guide effective research and development of PSC-derived materials for academic and industrial applications.

## 1. Introduction

Growing cell-based therapeutics utilizing pluripotent stem cells (PSCs), mesenchymal stem cells (MSCs), hematopoietic stem cells (HSCs), or chimeric antigen receptor T (CAR-T) cells offer novel disease treatment approaches. The use of PSCs, represented by induced pluripotent stem cells (iPSCs) and embryonic stem cells (ESCs), opens the door for more precise disease modelling, drug screening, and regenerative medicine [1]. The use of iPSCs, which overcomes the ethical issues associated with ESCs, especially broadens prospects for disease- or, even, patient-specific models and autologous cell treatments. Currently, the efficacies of PSC-derived cell therapy products are being assessed in over 30 clinical trials worldwide [2].

However, several issues surrounding the use and application of PSCs must be resolved to take advantage of their potential benefits. Among the hundreds of types of human cells [3], most have never been properly differentiated from PSCs or maintained in vitro, restricting their research and industrial use. Even if differentiation protocols are available, comparisons of PSC-derived cells and reference cells, such as primary cultures, often exhibit different cell states and functions. Although these differences are partly resolved by advanced cultivation technologies (e.g., three-dimensional (3D) culture, co-culture of different types of cells, or organoid construction), further improvements are anticipated. Moreover, for the broader use of PSC-based therapy and regenerative medicine, both stable mass production of PSC-derived materials and cost reduction should be achieved [4]. In addition, to overcome safety issues of heterogeneity, genetic instability, and tumorigenicity that sometimes hamper smooth PSC therapeutic applications [5,6,7], the whole process from PSC establishment to differentiation requires further screening and optimization.

To appropriately assess a cell-differentiation process, there are considerable requirements to evaluate in addition to the general process of cell expansion. During cell expansion, cells grow and increase through cell division; thus, it is critical to maintain cell quality and condition so as not to disturb homologous self-renewal [8]. Therefore, for the expansion of stem cells or other cell types (e.g., to produce a recombinant protein or antibody), cells are cultivated in specific optimized media and conditions. In contrast, during cell differentiation, cells often divide heterogeneously [9], exhibit altered cell characteristics, and mature in a temporal manner. Moreover, during prolonged culture processes, cells mature but no longer increase in number; instead, unnecessary cells are excluded in some cases.

Therefore, in general, stem cell differentiation processes are divided into phases based on cell maturation stages, with different media and culture conditions used for each phase. Compositions of media and extracellular matrixes (ECM) have primarily been determined through optimization based on findings from research of in vivo embryonic development [10]. Recently, cytokines, chemical compounds, small molecules, and micro-RNAs have been utilized to control cell-lineage specification [11,12]. Additionally, novel culture methods and devices have further broadened PSC applications while offering a myriad of possible conditions to optimize [13].

As the necessities for screening and optimization of PSC differentiation multiply, so do time and costs for detailed or large-scale experiments, which can hamper productivity. At present, PSC culture is relatively expensive and requires diligent medium changes. In addition, recent advancements in culture technologies can prolong the cultivation period to months, this inevitably makes it difficult to confirm experimental reproducibility.

To circumvent such difficulties in cell culture optimizations, a number of design of experiments (DOE) approaches have been implemented for cell expansion and differentiation processes—including PSC technology [14,15]. DOE enables effective condition screening and optimization while reducing experimental runs by virtue of statistics. Since the first application of analysis of variance (ANOVA) toward improved crop yield by Fisher in the 1920s, various approaches have supported academic and industry researchers. Recently, the perceived utility and spread of user-friendly statistical software have supported DOE applications in biological experiments [16,17]. Among such studies, numerous reports from the field of fermentation that achieved efficient optimizations of bacterial growth and fungi growth, and improve final product yield, suggest great applicability of DOE for mammalian cell culture optimization [18,19,20,21].

The use of DOE is not only recommended for experimental efficiency, but also for the reliability of cell-derived material production processes required by governments and regulatory authorities. DOE methodologies are now standardized by the International Organization for Standardization (ISO), while authorities including the United States Food and Drug Administration (FDA) and European Medicines Agency (EMA) recommend using DOE for pharmaceutical product development [14,22]. Notably, the success of Quality by Design (QbD) concepts in medical fields has urged DOE-based process evaluations in cell production facilities [23,24,25].

In this review, we collect and summarize DOE investigations of mammalian cell cultivation processes to support readers incorporating DOE approaches for their own experiments. First, we briefly introduce major experimental designs and their characteristics. Then, we discuss previous articles assessing stem cells, Chinese hamster ovary (CHO) cells, other cell types, and miscellaneous cell culture-associated processes. Finally, we describe the limitations and issues to be resolved for more effective PSC expansion and differentiation.

## 2. DOE Approaches

**Figure 1:**
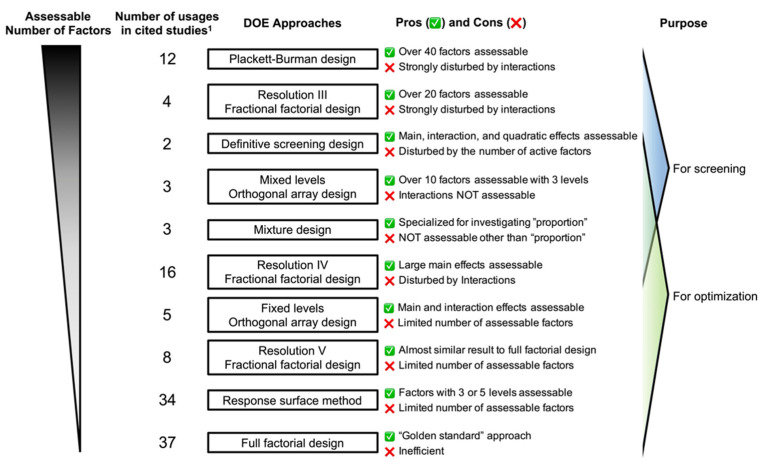
Pros and cons of each DOE approach. DOE, design of experiments. ^1^ Number of usages in cited studies were determined based on, in total, 124 DOE-based experiments.

In DOE applications, a trade-off between model accuracy and experimental efficiency is inevitable when estimating “main effects”, “interaction effects”, and “quadratic effects” of investigated factors for “response variables” (Figure 1). Response variables are the indicators measured and improved, e.g., marker gene/protein expression, cell yield, or cell purity. Main effects reflect the contribution of the investigated factor itself; interaction effects reflect synergistic or counteracting effects elicited by a combination of two or more investigated factors; quadratic effects reflect the curvature contributed by each investigated factor. Generally, specifying significant main effects should be prioritized over evaluating higher-order effects because main effects tend to be more robust and reproducible. In addition, three or more higher-order interactions are often neglected in DOE approaches. Compared with other scientific or industrial experiments, chemical or biological interactions largely hinder effective investigations because they are often unforeseeable and uncontrollable.

### 2.1. One Factor at a Time Approach

The most classical and simple experimental method is the “one factor at a time” (OFAT) approach. OFAT clarifies the main effect of a single factor at multiple levels using a moderate experimental scale. However, the accuracy and reproducibility of results are often inferior to those of other DOE approaches, and screenings easily end up with inefficient quasi-optimizations [26].

### 2.2. Full Factorial Design

Compared with OFAT, DOE enhances screening efficiency and result reproducibility by selecting optimal conditions through investigations of multiple factors and levels. Full factorial designs provide a clear estimation of the investigated response variables in an experimental space [27]. However, its practical use is limited to small experimental scales because investigation of more factors and levels requires a tremendous number of experimental runs.

### 2.3. Fractional Factorial Design

Fractional factorial designs are used to estimate main effects and interactions with a reduced number of experimental runs. By compromising the resolution of an assay [28], its experimental scale can be reduced to 1/2, 1/4, 1/8, 1/16, and so on of the original full factorial design. All main effects and two-order interactions remain estimable by “resolution V or more” designs to almost the same extent as full factorial designs, but the risk of missing optimal conditions increases as resolution decreases to “IV” or “III”. The Plackett–Burman design, another fractional factorial design, has been widely used for screening of media compositions owing to its relatively higher screening efficiency focusing on only large main effects. Because fractional factorial designs generally handle two-level factors, usages are limited to screening and further optimizations would be recommended for numerical factors possessing significant contributions.

### 2.4. Orthogonal Array Designs

The use of orthogonal array designs enables screening of multiple factors with multiple levels [29]. Fixed two-level (e.g., L_4_, L_8_, L_16_, and so on) or three-level (e.g., L_9_, L_27_, and so on) arrays are used to estimate all main effects and interactions of interest. Mixed-level arrays (e.g., L_12_, L_18_, L_36_, and so on) enable efficient identification of large main effects by ignoring interaction effects, based on the belief that controlling main effects is much easier and robust than controlling interactions and quadric effects [30]. Larger arrays offer extremely high experimental efficiency and ready-made analytical formats (e.g., various ways of signal-to-noise ratio calculation and two-step optimizations, and so on) enable efficient screening and even optimized fine-tuning. In addition, robust parameter design (RPD), which intentionally includes a “noise factor” in experimental arrays, essentially focuses on enhancing quality stability. Especially, L_18_ array-based RPDs have been widely utilized in industrial fields for the product designs and manufacturing process designs, which should be robust to uncontrollable factors, such as differences in lot size and lot-to-lot differences [31].

### 2.5. Response Surface Method (RSM)

Key factors selected through DOE screenings can be further optimized through DOE optimizations, represented by the RSM. With RSM, main effects, interactions, and quadratic effects can be efficiently deduced, and the modeled response surface gives clear hints for further optimization. More logically, statistical approaches like steepest ascent analysis offer optimal conditions. Despite of its powerful modelling availability, because RSM requires three or five levels for each factor, the number of assessable factors would be limited practically. Another concern would be the effects of outlier data, missing values, and extremely high or low values, on model constructions, which restrict the experimental space to conservatively small.

### 2.6. Definitive Screening Design (DSD)

DSD is an emerging DOE that possesses fascinating merits [32]. With small experimental runs ((2 × number of factors) + 1), main effects can be defined, large interactions can be detected, and quadratic effects can be estimated. These features readily offer a response surface for factor selection and further optimization from the initial screening results. However, the use of DSD is especially recommended for the screening of novel factors with unknown effects because more than three active factors in the design fail to construct good models. In addition, other screening designs specialized for main effect detection, such as fractional factorial designs and orthogonal array designs, would more precisely identify factors possessing main effects.

### 2.7. Mixture Design

Another potent approach available for media improvement is mixture design, which is especially suitable for blending-ratio optimization. This method has been vigorously utilized in the food, beverage, and drug fields [33], and its use for cell culture improvements should facilitate optimization of any proportional investigation, e.g., ECM composition, co-culture cell ratio, or nutrient composition. Although the usages are limited to the ratio or proportional assessments, mixture designs offer response surface models and experimental design spaces could be set more flexibly than general RSM avoiding unnecessary experimental conditions.

### 2.8. Selection of DOE Approach

**Figure 2:**
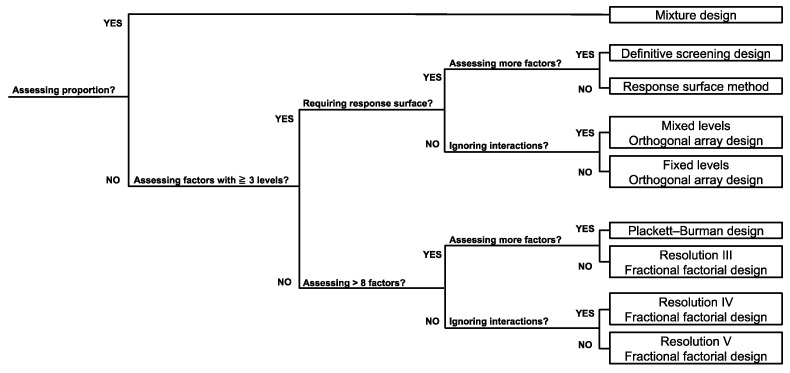
Decision tree for DOE selection.

From the major strategies described above, experimenters can choose and construct appropriate experimental designs to achieve specific goals (Figure 2). For efficient multifactorial screening, the use of low-resolution fractional factorial screenings, DSD, and mixed-level orthogonal array designs is recommended. Based on the screening results, optimal conditions can be chosen through fixed-level orthogonal array designs, high-resolution fractional factorial designs, full factorial designs, and RSM assessing a selected small number of factors. Above all, because it is crucial in DOE to reasonably select the response variables, factors, and levels of factors to be investigated, those settings should be fully discussed after a vigorous literature research for marked process improvement.

## 3. Stem Cell Expansion and Differentiation

In this chapter, we select DOE reports investigating PSC, MSC, or HSC cultivations. These representative stem cells, in common, require efficient cell expansion and differentiation for advanced research and application. Although the required media components are totally different, DOE facilitates efficient screening and optimization as a universal framework whose methodology can be applied for future investigations of any cell culture paradigm. In each section, commonly used DOE and distinguished works are briefly introduced.

### 3.1. PSC Expansion and Differentiation

For investigations of PSC expansion (Table 1), full factorial screenings have been used to select cytokines and ECM that maintain ESC pluripotency [34] and optimize bioreactor parameters for aggregate ESC cultures [35]. RSM was used to investigate the basic cell signaling of ESCs under hypoxic conditions [36], develop novel human iPSC (hiPSC) maintenance media [37], and increase the yield of automated ESC [38] and microcarrier PSC culture systems [39]. In these RSM studies, models were developed for optimizations of cell growth rate, colony-forming efficiency, population doubling, and cell yield of PSCs.

For investigations of PSC differentiation (Table 2), full factorial designs were used to screen additives during endoderm induction from embryoid bodies [40]; optimize ECM and cytokines during hepatic, cardiac, or mesodermal progenitor lineage induction [41,42,43]; optimize additive doses for retinal organoid production [44]. Fractional factorial screenings have also been used for definitive endoderm differentiation [45]. Mixed-level orthogonal arrays were used to screen cytokines for choroidal endothelium differentiation [46] and optimize additive doses for four distinct types of endodermal cell induction [47]. RSM was used to optimize hydrogel peptide concentrations for neural progenitor maturation [48], optimize the ECM composition for cardiomyocyte differentiation [49], and screen nutrient compositions driving trilineage specification from hiPSCs based on the response surface models for endodermal, mesodermal, and ectodermal protein expressions [50].

During early in vivo development, human embryos are exposed to dramatic environmental changes upon maternal blood perfusion [51]. From this perspective, Esteban et al. constructed a full factorial 3^3^ RSM design space composed of O_2_, glucose, and pyruvate to assess how the absence and presence of these key nutrients affects trilineage specification upon spontaneous differentiation of hiPSC. By assessing key marker gene sets, they revealed that O_2_ deprivation promoted ectodermal differentiation; O_2_ deprivation and low glucose synergistically promoted mesodermal differentiation; high O_2_ and low glucose synergistically promoted endodermal differentiation. The temporal transition of the generated response surfaces clearly showed the different contributions of the three factors.

Although 3D cultures strongly facilitate PSC-derived cell maturation compared with two-dimensional cultures, the culture processes involved are relatively complicated and need longer culture periods, which require detailed condition screenings. Jung et al. showed that interactions between three ECM components (collagen I, fibronectin, and laminin 111) largely contributed to cardiac troponin T protein expression and finely tuned the matrix composition via an RSM with just 15 runs [49]. Importantly, the optimal cell matrix reproducibly enhanced cardiomyocyte differentiation.

To investigate definitive endoderm differentiation and patterning, we constructed an L_18_ array design including whole anterior-posterior endoderm by inputting eight cell signaling modifiers, thereby increasing the screening efficiency 243-fold (18 runs vs. 4374 runs) [47]. RNA expression of 18 end products seemed like “melting pots”, in which some achieved specific differentiation and others produced mixtures of cell lineages. These varied results enabled the selection of optimal conditions for upregulating desirable genes, while downregulating undesirable ones for specific anterior foregut, hepatic, pancreatic, and mid-hindgut cell inductions. Following initial screening of two hiPSC lines, the constructed protocols were successfully applied to an additional five hiPSC lines.

Another mixed-level orthogonal array design approach reported by Songstad et al. screened five cell signaling proteins that facilitate differentiation of hiPSC-derived embryoid bodies into choroidal endothelial cells [46]. An L_12_ array facilitated the five-factor screening 2.7-fold (12 runs vs. 32 runs) and identified positive contributions. Notably, the screening results revealed a significant contribution of connective tissue growth factor (CTGF) through cell signal inhibitor analysis.

Survival and differentiation efficiency of neural cells largely depend on the ECM and its peptide compositions. Lam et al. developed a novel hydrogel matrix and optimized the gel formulation by investigating the ratio of three adhesion peptide components via repeated RSM optimizations [48]; the results revealed positive or negative interactions between the components. Along with component optimization, both cell survival and cell spreading efficiency were improved, and immunostaining and cell sorting analysis verified significant neuronal maturation.

As shown in Table 1 and Table 2, most DOE studies investigating PSCs focused on full factorial or RSM optimization. This suggests a need and opportunities for wider screening of uninvestigated factors to overcome technical limitations of PSC expansion and differentiation. Although investigations of ECM are likely more difficult than those of soluble factors, the use of ECM array platforms has enabled high-throughput full factorial screening of up to 128 combinations with replicates [41,42,43]. Incremental iPSC differentiation studies from the late 2010s presage further DOE exploitations in this field.

### 3.2. MSC and HSC Expansion, and Differentiation

For investigations of MSCs (Table 3), full factorial screenings were used to identify key parameters for automated expansion [52] and screen additives for chondrogenic differentiation [53]. Fractional factorial screening was used to develop a serum-free expansion medium [54]. Orthogonal arrays were used to optimize primary MSC culture conditions from umbilical cord blood, readily identified the significant contributions of initial cell density and cytokine doses for MSC growth [55]. RSM was used to optimize osteoblast and tenocyte differentiations, which offered models for marker RNA expression levels [56,57].

For clinical use of cell-derived materials, one of the big challenges is the development of chemically defined, animal origin-free cell culturing processes. After pre-screening of over 15 compounds, Liu et al. chose and screened four additives using a resolution IV 2^4−1^ fractional factorial design, and examined the viability of human cord blood MSCs [54]. A mere eight runs of cultivation defined a serum-free medium capable of yielding MSCs possessing the same growth ability and adipogenic, chondrogenic, and osteogenic differentiation potentials as those maintained in serum-containing media.

For efficient differentiation of human MSCs into chondrocytes, Jakobsen et al. screened five cytokines with 2^5^ full factorial design [53]. For each of the 32 cultivation runs, the expression of 364 chondrogenic genes was analyzed. The gene set contained both desirable articular cartilage markers and undesirable bone or adipose markers, thus enabling detection of marked contributions of transforming growth factor β1 (TGFβ1) and dexamethasone (DEX) to chondrogenesis.

For investigations of HSC (Table 4), full factorial designs were used to screen cytokines for long-term culture-initiating cells and colony-forming cell bifurcation [58,59,60], as well as platelet production [61]. Fractional factorial designs were used to screen the cytokines relative to HSC expansion [62,63] and its lineage commitments toward erythroid, granulocyte, megakaryocyte, and dendric cell [64,65,66,67]. RSM was used for the detailed optimization of HSC expansion [68] and to identify long-term culture-initiating cells or colony-forming cell bifurcation based on cell yields [69] and megakaryocyte induction was optimized through the modeling of cell yield, cell expansion ratio, and platelet production [70]. The resulting cytokine usages, lineage commitments, and DOE approaches for HSC investigations were diligently reviewed by Lim et al. [71].

To optimize media components for HSCs and their derivatives, Yao et al. reported numerous DOE results and employed systematic approaches by combining fractional factorial screening and steepest ascent. In their 2003 report, Yao and colleagues isolated HSCs from cord blood and screened numerous cytokines and serum components by low-resolution fractional factorial screenings [63]. The established medium expanded HSCs, colony-forming cells, and white blood cells more efficiently than other published results. Similar strategies were applied for direct HSC expansion from umbilical cord blood mononuclear cells to omit the HSC-isolation phase, which increased the experimental efficiency up to 64-fold (16 runs vs. 1024 runs) [64]. They also achieved megakaryocyte or platelet production [65] and potent antigen-presenting dendric cell differentiation from their HSCs [66] by applying fractional factorial screenings.

.

Differentiation of HSCs has been thoroughly investigated by DOE. The significant factors chosen from low-resolution fractional screening, including Plackett–Burman designs, seem to reproducibly contribute to the desired cell expansions and differentiations, probably due to their larger main effects compared with the interaction effects brought about by other factors. Notably, the wide varieties of target cell lineages and cytokines investigated suggest the applicability of multifactorial screening to enhance differentiation.

## 4. CHO Cell Expansion

CHO cell lines are exemplary mammalian cells whose culture processes have been thoroughly investigated using various DOE (Table 5). Indeed, detailed media optimizations utilizing DOE have been reported for effective recombinant protein and antibody production processes. We collected DOE investigations evaluating cell yield and/or viability as response variables [72,73,74,75,76,77,78,79,80,81,82,83,84,85,86,87,88,89,90,91,92,93,94,95], or selecting desirable clones [96,97]. 

Although the base media used for PSC differentiation mostly depend on commercial media, such as RPMI, Eagle’s, and Ham’s media with some supplements, nutrient levels in CHO culture media have been optimized through detailed investigations, sometimes for each clone. Torkashvand et al., achieved a 1.7-fold increase of monoclonal antibody (mAb) titer by screening 19 amino acids in 20 runs with a Plackett–Burman design, followed by RSM dose optimization of the four key amino acids [76]. Obvious inhibitory effects arising from some unnecessary amino acid additions highlight the importance of dedicated evaluations of base media for cell quality improvements.

Selecting the right cell strain or clone can resolve difficulties in later research and developmental stages, in some cases because some human PSC characteristics originate from the process used to establish the clone [98,99]. In a CHO cell line-development process, Mora et al. employed factorial DOE to feed 10 CHO clones with 24 different feed plans [73]. This screening revealed that the clones could be divided into early and late responders, which differed in their peak timing for molecule production. On the basis of the screening results, they modified the feeding strategy and improved the molecule titers of seven investigated clones (up to 34% increase), while reducing about 40% of hands-on time for culture maintenance by skipping unnecessary media changes.

CHO cells originally maintained adherently in the presence of serum are often adapted to serum-free cell suspension culture to improve productivity and quality, especially in industry. This adaptation process was optimized by Wu et al., who screened five additives employing DSD in just 15 experimental runs [95]. Consequently, with the established medium condition, the adaptation process that conventionally took 66 days was successfully shortened to just 27 days.

Screening of wider design spaces with more data points can be investigated through mixture design approaches. Jordan et al. prepared 10 different nutrient cocktails, each containing different combinations of 20 amino acids, and further systematically blended the 10 cocktails at various combinations and ratios. From the resulting 192 media, the medium generating the highest mAb titer was chosen [97]. More detailed statistical analysis revealed the limiting dose of each amino acid, suggesting that further optimization of medium components is possible on the basis of the accumulated data. Likewise, Rouiller et al. included 43 nutrients in 16 cocktails, and mixed them to obtain 376 media [93]. Some media increased the mAb titer up to 1.4-fold compared with the control condition, and further improvement was predicted through the identification of key components.

In addition to screening cytokines, the base medium components for CHO cell culture have been minutely investigated. Such approaches should be applied not only for PSC expansion processes, but also to optimize base media for differentiation of cells to increase yield and/or purity of the end material. Screening efficiency was further increased by regarding groups of nutrients as a factor, based on their roles in metabolic pathways. Notably, the use of mixed-level orthogonal array designs, mixture designs, and DSDs for cell culture have great potential to further accelerate media development because of their high screening efficiency.

## 5. Other Cell Expansion, Cell Differentiation, and Cell-Material Development Processes

Cell isolation and/or selective expansion of target cells (Table 6) also requires intricate screening, and this process needs to be robust enough for reproducible utilization for different cell origins and donors. To improve human cell yields, culture conditions for pancreatic duct cells were assessed by full factorial design [100], or co-culture of intestinal Caco-2 cells and goblet HT29-MTX cells [101], umbilical vein endothelial cells [102], cytotoxic T lymphocytes [103], prostate cancer cells [104], and immortalized erythroblasts [105] were investigated by RSM modeling of cell numbers. In addition to these human cell types, Vero cells [106,107] and murine cells [108,109,110] have been assessed by fractional factorial and orthogonal array designs.

To improve cell differentiation (Table 7), human chondrocytes were differentiated into cartilage [111,112], human periosteum-derived cells and osteosarcoma cells were investigated for skeletal tissue development [113], human adipose-derived stromal cells and murine embryonic fibroblasts were differentiated into osteoblasts [114,115], human hepatoma cells were differentiated into hepatocytes [116], and mouse pluripotent embryonic carcinoma were differentiated into neuronal cells [117]. In these RSM optimizations, response surfaces of cell spreading, osteogenic markers [115], and metabolites [116] were obtained.

The use of DOE is not limited to cell cultivation and can also be applied for other cell-related purposes (Table 8), such as cell storage, cell transportation, and related materials and devices, which are especially important issues for industrial cell usage. The refrigerated storage conditions for retinal pigment epithelial cells [118] and epithelial cell sheets [119] were optimized through full and fractional factorial screening. In addition, the virus inactivation process of Vero cells [120] was investigated using an orthogonal array. Optimal freezing conditions for CHO and human embryonic kidney (HEK) cells were identified by RSM through the modelling of the first doubling time after the cell thawing [121].

For prostate cancer cell expansion toward vaccine development, Zhao et al. screened 16 compounds in 20 runs using the Plackett–Burman approach [117]. This screening indicated that epidermal growth factor (EGF), fibroblast growth factor (FGF), and linoleic acid had the highest positive effects on cell yield. The best doses of these additives were determined by RSM in another 20 runs of experiments. The established medium was confirmed to support prostate cancer cell growth equal to or greater than serum-containing medium.

During in vitro chondrocyte expansion, some mature chondrocytes undesirably dedifferentiate into immature fibrous chondrocytes. This obstacle to chondrocyte amplification is overcome through redifferentiation culture, in which expanded fibrous chondrocytes are again matured while avoiding hypertrophic differentiation. This cell bifurcation was inspected by two different DOE approaches. First, Enochson et al. optimized the redifferentiation medium by assessing the gene expression of mature and immature chondrocyte markers [112]. Five-factor fractional factorial screening (2^5−1^) extracted contributors, while subsequent RSM optimizations established the medium, to induce histologically better micromass formation than conventional medium. Throughout the investigation, gene expression of collagens was not ideally controlled; thus indicating that collagen expression might be regulated by other cell signaling pathways and that further screening of collagen regulators could lead to quality improvement. Liu et al. screened 12 candidate regulators for auricular chondrocyte redifferentiation by monitoring glycosaminoglycan accumulation as a response variable by 2^12−4^ fractional factorial design [111], which increased screening efficiency 16-fold (256 runs vs. 4096 runs). A combination of three cytokines was found to promote glycosaminoglycan accumulation while preventing hypertrophic differentiation. Efficacy of the optimized medium was validated by redifferentiation of chondrocytes from different sources (articular and rib), mechanical property analysis of 3D-cultured cartilage pellets, and pellet implantation into nude mice.

In this chapter, we confirmed that DOE facilitated optimization of cell culture processes, regardless of the cell lineages involved. Although an enormous number of factors were incorporated in DOE for different purposes, DOE successfully identified positive factors and optimal conditions. The versatility of DOE promises to accelerate PSC-derived material research and development in any situation.

## 6. Concluding Remarks

The presented efforts adopting DOE approaches in mammalian cell culture confirm that DOE-based screening and optimization of stem cell culture facilitates novel differentiation protocol developments and strategic evidence accumulation. Moreover, technical hurdles arising from different cell characteristics of donors or clones might be overcome by the construction of robust universal media components or donor-specific customized media through high-throughput optimization. In particular, multifactorial screening has good opportunities to reveal novel cell signaling regulation, which is not only important for its biological meaning, but also desirable for stable and high-quality cell-differentiated materials.

There is still room for more effective utilization of DOEs, especially for PSC investigations. Indeed, because most previous studies were based on closed settings with full factorial or RSM designs, rather than open for novel factor searching [122], the high experimental efficiency of DOE is not fully utilized. In addition, because most PSC studies were designed to assess cell-signaling modifiers, nutrient composition could also be improved, as evaluated in CHO and other cell investigations [123]. Emerging metabolome analysis and online monitoring of medium compositions along the differentiation process should offer hints for further multifactorial screening. The significant contributions of basal media composition on PSC lineage commitment [50] suggest that further addition or removal of energy sources can improve the quality and/or yield of PSC and PSC-derived materials.

The timing and duration of additive treatments seem to be poorly investigated, although differentiating cells mature during the cultivation process. For instance, in the course of endoderm differentiation, the timing and duration of retinoic acid exposure significantly affects lineage commitment both in vivo and in vitro [124]. To assess time-dependent regulation of cell signaling, three or more multilevel screenings using orthogonal arrays or DSDs might be more suitable. However, because incorporation of time-related factors in DOEs tends to enlarge interaction effects, experimental plans should be carefully reviewed.

Another underexplored time-related issue is simultaneous investigation of media components from different cultivation phases. For example, if a differentiation process consists of three phases, the first, second, and third phases are often sequentially developed, and then a problematic phase might be refined. This “one phase at a time” approach leads to quasi-optimal or frail conditions in the same manner as OFAT [15]. Particularly for cell differentiation processes, it is strongly supposed that the cell status (e.g., epigenetic regulation) in earlier phases largely influences the cell-lineage commitment of later phases; thus, interactions between factors used in different phases would have significant effects. DOE investigations of whole-process development methodologies, such as the studies summarized in Section 6 or reviewed elsewhere [125,126], offer hints for better experimental planning.

To screen numerous conditions appropriately, novel devices and platforms (such as those used for ECM optimizations) can decrease experimental cost, human error, and statistical experimental error. Utilization of robotics should especially be considered when incorporating many factors or implementing a large-scale mixture design, an intricate media preparation or media-change scheme [97,127]. The use of robots can further promote sequential optimizations through DOE planning, subsequent cell culture, cell characterization by omics analysis and/or cell sorting, and validation of candidate optimal protocols without human intervention. Those acquired data should be particularly suitable for further in silico modeling of cell culture processes [128,129,130].

The use of DOE has no drawbacks if the experimenters have enough knowledge and skills in the research field. Because DOE prompts research activities whose concrete utilities have been ubiquitously elucidated, its use and understanding in academia, industry, and administrative authorities are more essential than ever. We are certain that wider DOE utilization will reveal new biological insights and novel solutions to establish more efficient cell differentiation processes for further cell material applications.

## Figures and Tables

**Table 1:** DOE studies investigating PSC expansion.

Cells	Experimental Design	Number of Factors	Factors	Year	Ref.
Murine ESC	Full factorial 2^4^	4	LIF, FGF4, Fibronectin, Laminin	2004	[34]
	Full factorial 2^3^	3	FGF4, Fibronectin, Laminin		
Human ESC	Full factorial 3^2^	2	Seeding density, Agitation speed	2014	[35]
Murine ESC	RSM	3	CHIR99021, LIF, PD0325901	2012	[36]
Human ESC	RSM	4	Seeding density, Media volume, Media exchange time, Duration between passages	2013	[38]
Human iPSC	RSM	2	bFGF, NRG1β1	2015	[37]
Human iPSC	RSM	2	Seeding density, Agitation speed	2016	[39]

Abbreviations: LIF, leukemia inhibitory factor; FGF4, fibroblast growth factor 4; bFGF, basic fibroblast growth factor 2; NRG1β1, neuregulin 1 β1.

**Table 2:** DOE studies investigating PSC differentiation.

Cells	Purpose	Experimental Design	Number of Factors	Factors	Year	Ref.
Murine ESC	Endodermal differentiation	Full factorial 2^5^	5	Glucose, Insulin, bFGF, Retinoic acid, EGF	2004	[40]
		Full factorial 3^2^	2	Retinoic acid, EGF		
Murine ESC	Hepatocyte differentiation	Full factorial 2^5^	5	Collagen I, Collagen III, Collagen IV, Laminin, Fibronectin	2005	[41]
Murine ESC	Cardiac cell differentiation	Full factorial 2^5^	5	Collagen I, Collagen III, Collagen IV, Laminin, Fibronectin	2008	[42]
		Full factorial 2^4^	4	Wnt3a, Activin A, BMP4, FGF4		
Human iPSC	Mesodermal progenitor differentiation	Full factorial 2^7^	7	Collagen I, Collagen III, Collagen IV, Collagen V, Laminin, Fibronectin, Vitronectin	2015	[43]
Human iPSC	Retinal organoid differentiation	Full factorial 2^5^	5	Initial cell density, 1-Thioglycerol, BMP4, KSR, Lipids	2018	[44]
		Full factorial 2^4^	4	Initial cell density, CHIR99201, BMP4, SU5402		
Human iPSC	Definitive endoderm differentiation	2^4−1^ Resolution IV	4	Activin A, GDF8, Wortmannin, CHIR99201	2020	[45]
Human iPSC	Choroidal endothelium cell differentiation	L_12_	5	CTGF, CTNNB1, SHC1, TWEAKR, VEGFB	2017	[46]
Human iPSC	Four endodermal cell differentiation	L_18_	8	Retinoic acid, CHIR99201(early phase), bFGF(later phase), Sodium butyrate, bFGF(early phase), CHIR99201(later phase), (LDN193189, BMP4), A-83-01	2021	[47]
Human iPSC-derived	Mature neuron differentiation	RSM	3	RGD, YIGSR, IKVAV ^1^	2015	[48]
neural progenitor cell		RSM	2	RGD, IKVAV ^1^		
		RSM	2	RGD, IKVAV ^1^		
Murine iPSC	Cardiomyocyte differentiation	Full factorial 2^3^	3	Collagen I, Laminin, Fibronectin	2015	[49]
		RSM	3	Collagen I, Laminin, Fibronectin		
		Full factorial 2^3^	3	Collagen I, Fibronectin, TSP1		
Human iPSC	Trilineage bifurcation	RSM	3	O_2_ tension, Glucose, Pyruvate	2021	[50]

Abbreviations: bFGF, basic fibroblast growth factor 2; EGF, epidermal growth factor; BMP4, bone morphogenetic protein 4; FGF4, fibroblast growth factor 4; KSR, KnockOut™ Serum Replacement; GDF8, myostatin or growth differentiation factor 8; CTGF, connective tissue growth factor; CTNNB1, β catenin; SHC1, steroid receptor coactivator homology 2 domain-containing transforming protein 1; TWEAKR, TNF-related weak inducer of apoptosis receptor; VEGFB, vascular endothelial growth factor B; TSP1, thrombospondin.^1^ RGD, YIGSR, and IKVAV are peptides containing the indicated amino acid residues.

**Table 3:** DOE studies investigating MSC expansion and differentiation.

Purpose	Experimental Design	Number of Factors	Factors	Year	Ref.
MSC expansion	Full factorial 2^4^	4	Seeding density, Fetal calf serum, Media volume, Culture time	2008	[52]
Chondrocyte differentiation	Full factorial 2^5^	5	TGFβ1, BMP2, DEX, FGF2, IGF1	2014	[53]
MSC expansion	2^4−1^ Resolution IV	4	Hydrocortisone, bFGF, Human albumin, SITE supplement ^1^	2007	[54]
MSC expansion	L_8_	4	Seeding density, Cytokines ^2^, Serum, Stromal cells	2009	[55]
	L_8_	6	SCF, TPO, FL, IL-3, GM-CSF, G-CSF		
Osteoblast differentiation	RSM	4	Culture duration, O_2_ tension, Seeding density, Two media ^3^	2011	[56]
Tenocyte differentiation	RSM	2	TGFβ3, Culture days	2020	[57]

Abbreviations: TGFβ1, transforming growth factor β1; BMP2, bone morphogenetic protein 2; DEX, dexamethasone; FGF2, fibroblast growth factor 2; IGF1, insulin-like growth factor 1; bFGF, basic fibroblast growth factor 2; SCF, stem cell factor; TPO, thrombopoietin; FL, FMS-like tyrosine kinase 3 ligand; IL-3, interleukin-3; GM-CSF, granulocyte macrophage-colony stimulating factor; G-CSF, granulocyte-colony stimulating factor; TGFβ3, transforming growth factor β3.^1^ SITE supplement contains sodium selenite, bovine insulin, human transferrin, and ethanolamine.^2^ Cytokines contain stem cell factor, FL, TPO, IL-3, G-CSF, and GM-CSF.^3^ Two media are α-MEM supplemented with ascorbate-2-phosphate or α-MEM supplemented with ascorbate-2-phosphate, DEX, and β-glycerophosphate.

**Table 4:** DOE studies investigating HSC expansion and differentiation.

Purpose	Experimental Design	Number of Factors	Factors	Year	Ref.
LTC-IC and CFC bifurcation	Full factorial 2^5^	5	FL, SF, IL-3, IL-6, (G-CSF, NGFβ)	1996	[58]
LTC-IC and CFC bifurcation	Full factorial 2^3^	3	FL, SF, IL-3	1997	[59]
LTC-IC and CFC bifurcation	Full factorial 2^6^	6	FL, SF, IL-3, (IL-6, sIL-6R), TPO, IL-1	1998	[60]
Megakaryocyte and platelet differentiation	Full factorial 2^4^	4	SCF, IL-3, IL-6, IL-9	2013	[61]
HSC expansion	2^9−5^ Resolution III	9	TPO, IL-3, SCF, FL, G-CSF, GM-CSF, IL-6, sIL-6R, EPO	2003	[62]
	2^4−1^ Resolution IV	4	TPO, IL-3, SCF, FL		
	2^8−4^ Resolution IV	8	Albumax, BSA, TF, Glutamine, Hydrocortisone, Peptone, 2-ME, Insulin		
	2^4^	4	BSA, Insulin, TF, 2-ME		
	2^7−3^ Resolution IV	7	TPO, IL-3, SCF, FL, G-CSF, GM-CSF, IL-6		
HSC expansion	Full factorial 2^4^	4	BSA, Insulin, TF, 2-ME	2004	[63]
	2^10−6^ Resolution III	10	TPO, IL-3, SCF, FL, IL-11, IL-6, GM-CSF, G-CSF, SCGF, HGF		
Erythroid cell, granulocyte, and megakaryocyte differentiation	2^7−3^ Resolution IV	7	FL, SCF, IL-3, (MGDF, G-CSF), IL-11, IL-6, EPO	2001	[64]
	Full factorial 2^4^	4	IL-3, IL-11, IL-6, EPO		
Megakaryocyte differentiation	2^8−3^ Resolution IV	8	TPO, IL-3, SCF, FL, IL-11, IL-6, GM-CSF, IL-9	2009	[65]
Dendritic cell differentiation	2^8−4^ Resolution IV	8	SCF, FL, IL-1β, GM-CSF, TNFα, IL-4, IL-6, TGFβ1	2019	[66]
	2^5−1^ Resolution V	5	SCF, FL, IL-1β, GM-CSF, TNFα		
HSC differentiation ability	2^5−1^ Resolution V	5	SCF, FL, TPO, SDF-1, Fucoidan	2011	[67]
	Full factorial 2^3^	3	SCF, FL, TPO		
HSC expansion	RSM	4	SCF, FL, TPO, LIF	2010	[68]
LTC-IC and CFC bifurcation	Full factorial 2^5^	5	IL-11, SF, FL, TPO, Temperature	2002	[69]
	RSM	3	IL-11, SF, FL		
Megakaryocyte and platelet differentiation	Plackett–Burman	11	SCF, FL, IL-11, MIP-1α, IL-1α, IL-1β, IL-8, IFN-γ, VEGF, MCP-1, β-thromboglobuline	2005	[70]
	Plackett–Burman	9	IL-9, IL-8, IL-6, IL-1α, IL-1β, SCF, FL, MIP-1α, IFN-γ		
	2^5−1^ Resolution V	5	SCF, FL, IL-6, IL-9, EPO		
	Full factorial 2^4^	4	SCF, FL, IL-6, IL-9		
	RSM	4	TPO, SCF, IL-6, IL-9		

Abbreviations: FL, FMS-like tyrosine kinase 3 ligand; SF, steel factor; IL-3, interleukin-3; IL-6, interleukin-6; G-CSF, granulocyte-colony stimulating factor; NGFβ, nerve growth factor β; sIL-6R, soluble IL-6 receptor; TPO, thrombopoietin; IL-1, interleukin-1; SCF, stem cell factor; MGDF, megakaryocyte growth and development factor; IL-11, interleukin-11; EPO, erythropoietin; GM-CSF, granulocyte macrophage-colony stimulating factor; TF, transferrin; 2-ME, 2-mercaptoethanol; SCGF, stem cell growth factor α; HGF, hepatocyte growth factor; IL-1β, interleukin-1β; TNFα, tumor necrosis factor α; IL-4, interleukin-4; TGFβ1, transforming growth factor β1; SDF-1, stromal cell-derived factor-1; MIP-1α, macrophage inhibitory protein-1α; IL-1α, interleukin-1α; IL-8, interleukin-8; IFN-γ, interferon γ; VEGF, vascular endothelial growth factor; MCP-1, monocyte chemoattractant protein-1.

**Table 5:** DOE studies investigating CHO cell expansion.

Experimental Design	Number of Factors	Factors	Year	Ref.
Full factorial 2^3^	3	Glucose, Glutamine, Inorganic salts	2004	[72]
Full factorial 2^5^	5	Feed volume at days 3, 5, 7, 10, and 12	2019	[73]
2^5−1^ Resolution V	5	Sodium hypoxanthine-thymidine, Antioxidant, ITS ^1^, Fatty acids supplement, Polyamines supplement	2006	[74]
2^4−1^ Resolution IV	5	Amino acid feed, Glucose Feed, Temperature, pH	2011	[96]
Full factorial 3^1^ × 2^2^	3	Glucose feed, Temperature shift, pH control frequency		
Plackett–Burman	20	BSA, Transferrin, Insulin, Sodium pyruvate, Putrescine, Glucose, Ala, Arg, Asn, Asp, Cys, Gln, Glu, Gly, Ser, Met, (Pro, His, Hydroxyproline), (Thr, Val, Ile), (Leu, Trp, Lys), (Phe, Tyr)	1992	[75]
Plackett–Burman	4	Oleic acid, Linoleic acid, Cholesterol, (Choline, Ethanolamine)	1995	[76]
Plackett–Burman	21	Ala, Arg, (Asn, Asp), Cys, Gln, Glu, Gly, Ser, Met, (Phe, Tyr), (Thr, Val, Ile), (Leu, Trp, Lys), (Pro, His), Insulin, Transferrin, Ethanolamine, Pluronic F68, Phosphatidylcholine, Putrescine, Linoleic acid, Hydrocortisone	1998	[77]
Plackett–Burman	21	Ala, Arg, (Asn, Asp), Cys, Gln, Glu, Gly, Ser, Met, (Phe, Tyr), (Thr, Val, Ile), (Leu, Trp, Lys), (Pro, His), Insulin, Transferrin, Ethanolamine, Pluronic F68, Phosphatidylcholine, Hydrocortisone, Sodium selenite, Glutathione	1999	[78]
Plackett–Burman	21	Ala, Arg, (Asn, Asp), Cys, Gln, Glu, Gly, Ser, Met, (Phe, Tyr), (Thr, Val, Ile), (Leu, Trp, Lys), (Pro, His), Sodium selenite, Insulin, Transferrin, Hydrocortisone, Ethanolamine, Phosphatidylcholine, Glutathione, Pluronic F68	1999	[79]
RSM	2	Glucose, Gln	2005	[80]
RSM	2	Glucose, NaCl		
2^7−3^ Resolution IV	7	Insulin, Meat peptone, Yeast extract, SerEx, BSA, Linoleic acid–BSA, Dextran sulfate	2006	[81]
RSM	2	Insulin, SerEx		
RSM	5	Gln, Essential amino acids supplement, Non-essential amino acids supplement, ITS ^1^, Lipids	2007	[82]
RSM	3	Yeastolate, Soy, Wheat	2009	[83]
Plackett–Burman	17	Ethanolamine, Sodium selenite, Putrescine, Hydrocortisone, Lipids, Sodium pyruvate, Ascorbic acid, Glutathione, Choline chloride, D-calcium pantothenate, Folic acid, Niacinamide, Pyridoxine-hydrochloride, Riboflavin, Thiamine hydrochloride, Cyanocobalamin, I-inositol	2013	[84]
RSM	3	Lipids, Putrescine, Ammonium ferric citrate		
RSM	3	Temperature, pH, Seeding density, Culture duration	2013	[85]
RSM	3	Glucose, Asn, Gln	2015	[86]
Plackett–Burman	19	19 amino acids (Gln excluded)	2015	[87]
RSM	4	Asp, Glu, Arg, Gly		
RSM	3	pH, O_2_ tension, CO_2_ tension	2017	[88]
2^8−4^ Resolution IV	8	8 kinds of commercial supplements	2020	[89]
RSM	4	4 kinds of commercial supplements		
Full factorial 2^3^	3	3 kinds of commercial supplements		
Plackett–Burman	8	Sodium selenite, Transferrin, Albumin, Insulin, Tocopherol, Tween 80, Fatty acids, Synthetic cholesterol	2019	[90]
Box–Behnken RSM	3	Transferrin, Insulin, Tween 80		
Plackett–Burman	15	Gln, Asp, Lys, Trp, Thr, Val, His, Vitamin B1, Thymidine, Deoxy-cytidine, 3-methyl-oxobutyrate, Deoxy-guanosine, Vitamin B6, Vitamin A, Arachidonate	2020	[91]
RSM	2	Thr, Arachidonate		
Mixture Design	6	Hexoses, Energy provider compounds	2007	[92]
Mixture Design	20	20 amino acids	2013	[97]
Mixture Design	43	19 amino acids (Gln excluded), Disodium phosphate, Magnesium sulfate, Calcium chloride, Myo-inositol, Sodium pyruvate, D-biotin, Choline Chloride, Folic acid, Niacinamide, D-pantothenic acid, Potassium chloride, Pyridoxine, Riboflavin, Thiamine, Ferric ammonium citrate, Vitamin B12, Hypoxanthine, Thymidine, Putrescine, Ethanolamine, Zinc sulfate, Cupric sulfate, Pluronic, Sodium selenite	2013	[93]
DSD	5	pH, Shifted temperature, Seeding density, Viable cell density at first feeding, Viable cell density at temperature shift	2019	[94]
DSD	6	DMEM fraction, Cellgro trace element A, Cellgro trace element B, Insulin, Ca^2+^, Mg^2+^	2021	[95]

^1^ ITS supplement contains sodium selenite, bovine insulin, and human transferrin.

**Table 6:** DOE studies investigating other cell expansion.

Cells	Experimental Design	Number of Factors	Factors	Year	Ref.
Human pancreatic duct cell	Full factorial 2^5^	5	bFGF, EGF, HGF, KGF, VEGF	2012	[100]
Vero	2^10−6^ Resolution III	10	(20 amino acids, Vitamin B1, Magnesium sulfate, Sodium phosphate), (Vitamins H, B2, and B9, Thymidine, Uracil, Xanthine, Hypoxanthine), (Vitamins B12, B3, and B7, Choline chloride, Pyridoxal), (Vitamins B3, B6, and BX, Putrescin), (Vitamins A, D2, and K3, Linoleic acids, Lipoic acids), (Deoxyribose, Adenine, Adenosine, Ethanolamine), (Plant and yeast extracts, EGF, Insulin), (Sodium citrate, Ferric chloride), (Glucose, Pyruvate), (Other)	2010	[106]
Murine hybridoma	2^9−4^ Resolution IV	9	Serum, Dissolved oxygen, Temperature, pH, Glucose, Glutamine, Lactate, Ammonium, Base medium concentration	1993	[108]
Murine myeloma	2^5−1^ Resolution V	5	pH, Temperature, Dissolved oxygen, Early/late feed regime, Seeding density	2000	[109]
Murine hybridoma	L_8_	4	Stirring speed, Fetal bovine or calf serum, Serum concentration, Glucose and glutamine supplement	2002	[110]
Vero	L_8_	4	Cytodex 1, Regulation of glucose, Initial glucose, Gln	2006	[107]
Caco-2 and HT29-MTX cells	L_18_	4	MEM or DMEM medium, Seeding time, Seeding density, and Caco-2/HT29-MTX ratio	2010	[101]
Human umbilical vein endothelial cell (HUVEC)	Full factorial 2^4^	4	RGDS, IKVAV, YIGSR, Q11 ^1^	2011	[102]
	RSM	3	RGDS, IKVAV, YIGSR ^1^		
Human peripheral blood mononuclear cell	2^4−1^ Resolution IV	4	Phosphatidyl choline, Polyamine supplement, Antioxidant supplement, Cholesterol	2010	[103]
	RSM	2	Polyamine supplement, Cholesterol		
Human prostate cancer cells	Plackett–Burman	16	Transferrin, Sodium selenite, Sodium L-ascorbate, Ferric citrate, L-glutathione, BSA, EGF, bFGF, Ethanolamine, Linoleic acid, Arachidonate, Thioglycerol, Hydrocortisone, Yeast hydrolysate, Penicillin-Streptomycin Solution, Succinic Acid	2017	[104]
	RSM	3	EGF, FGF, Linoleic acid		
Immortalized human erythroblast	2^9−4^ Resolution IV	9	BSA, EPO, Holo-transferrin, Hydrocortisone, Insulin, Fatty acid supplement, Lipid mixture solution, Non-essential amino acids supplement, SCF	2018	[105]
	RSM	3	BSA, EPO, Fatty acid supplement		

Abbreviations: bFGF, basic fibroblast growth factor 2; EGF, epidermal growth factor; HGF, hepatocyte growth factor; KGF, keratinocyte growth factor; VEGF, vascular endothelial growth factor; MEM, Minimum Essential Medium with Earle’s Salts; DMEM, Dulbecco’s Modified Eagle’s Medium; EPO, erythropoietin; SCF, stem cell factor.^1^ RGDS, IKVAV, and YIGSR are peptides containing the indicated amino acid residues. Q11 is the structural peptide used as control matrix.

**Table 7:** DOE studies investigating other cell differentiation.

Cells	Purpose	Experimental Design	Number of Factors	Factors	Year	Ref.
Mouse pluripotent embryonic carcinoma	Neuronal cell differentiation	Full factorial 2^3^	3	2D- or 3D-culture, IKVAV ^1^, ECM stiffness	2012	[117]
Human chondrocytes	Cartilage differentiation	2^12−4^ Resolution VI	12	BMP2, Insulin, IGF1, Testosterone, Parathyroid hormone, IL-1RA, Growth hormone, 17β-estradiol, Triiodothyronine, 1α-25-dihydroxy vitamin D3, FGF2, DEX	2007	[111]
Human chondrocytes	Articular chondrocyte differentiation	2^5−1^ Resolution V	5	TGFβ1, ASC, ITS, DEX, Linoleic acid	2012	[112]
		Full factorial 2^3^	3	TGFβ1, DEX, Glucose		
		Full factorial 2^2^	2	DEX, Glucose		
Human bone progenitor cells	Skeletal tissue development	2^5−1^ Resolution V	5	Medium volume, Seeding density, Human periosteum-derived cell or osteosarcoma cell, Seeding timing, Foamed titanium or 3D fiber-deposited titanium	2011	[113]
Human adipose-derived stromal cells	Osteoblast differentiation	12 × 12 Hadamard matrix ^2^	8	Two human adipose-derived stromal cells suppliers, Seeding density, DMEM/F12 or DMEM, Human platelet lysate or Fetal bovine serum, L-ascorbate-2-phosphate, β-glycerophosphate, DEX, BMP9	2019	[114]
Human hepatoma cell	Hepatocyte differentiation	2^7−4^ Resolution III	7	Human serum albumin, HGF, Oncostatin M, DEX, FGF4, EGF, Nicotinamide	2008	[116]
		RSM	3	Oncostatin M, HGF, FGF4		
Murine embryonic fibroblast cell	Osteoblast differentiation	RSM	2	Matrix stiffness, Collagen I	2010	[115]

Abbreviations: BMP2, bone morphogenetic protein 2; IGF1, insulin-like growth factor 1; IL-1RA, interleukin-1 receptor antagonist; FGF2, fibroblast growth factor 2; DEX, dexamethasone; BMP9, bone morphogenetic protein 9; HGF, hepatocyte growth factor; FGF4, fibroblast growth factor 4; EGF, epidermal growth factor. ^1^ IKVAV is a peptide containing the indicated amino acid residues.^2^ Hadamard matrix is a fixed level orthogonal array.

**Table 8:** DOE studies investigating other cell-related processes.

Cells	Purpose	Experimental Design	Number of Factors	Factors	Year	Ref.
Human retinal pigment epithelial cells	Cell storage condition	Full factorial 2^5^	5	Adenosine, Allopurinol, β-Glycerophosphate, L-Ascorbic acid, Taurine	2018	[118]
Human epithelial cell sheets	Cell storage condition	2^10−4^ Resolution IV	10	1% Glycerol, L-Ascorbic acid, Allopurinol, Sodium pyruvate, Adenosine, Taurine, L-Glutathione, Hydrocortizone, LiCl, Antimycin-A	2018	[119]
		2^10−4^ Resolution IV	10	0.75% Glycerol, 3% Glycerol, Icilin, Menthol, Dimethyl (S)-(−)-malate, Methyl pyruvate, N-Acetyl-L-Cys, Insulin, Acetovanillone, N-(2-Mercaptopropionyl)glycine		
		Full factorial 5^5^	5	L-Carnosine, Dimethyl sulfoxide, Fenoldopam mesylate, Glycerol, LIF		
		Full factorial 5^5^	5	Glycerol, Aspirin, Melatonin, Lactic acid, ATP		
Vero	Virus inactivation	L_9_	4	Temperature, Treatment time, pH, Ethanol	2019	[120]
CHO cell and HEK293	Cell freezing and refreezing condition	RSM	3	Freezing density, Dimethyloxide, Seeding density	2008	[121]

Abbreviations: LIF, leukemia inhibitory factor.
